# Nomogram Model to Predict Acute Kidney Injury in Hospitalized Patients with Heart Failure

**DOI:** 10.31083/j.rcm2508293

**Published:** 2024-08-20

**Authors:** Ruochen Xu, Kangyu Chen, Qi Wang, Fuyuan Liu, Hao Su, Ji Yan

**Affiliations:** ^1^Heart Failure Center, The First Affiliated Hospital of USTC, University of Science and Technology of China, 230001 Hefei, Anhui, China

**Keywords:** heart failure, acute kidney injury, nomogram, risk prediction

## Abstract

**Background:**

Acute kidney injury (AKI) is a common complication of acute heart failure (HF) that can prolong hospitalization time and worsen the prognosis. The objectives of this research were to ascertain independent risk factors of AKI in hospitalized HF patients and validate a nomogram risk prediction model established using those factors.

**Methods:**

Finally, 967 patients hospitalized for HF were included. Patients were randomly assigned to the training set (n = 677) or test set (n = 290). Least absolute shrinkage and selection operator (LASSO) regression was performed for variable selection, and multivariate logistic regression analysis was used to search for independent predictors of AKI in hospitalized HF patients. A nomogram prediction model was then developed based on the final identified predictors. The performance of the nomogram was assessed in terms of discriminability, as determined by the area under the receiver operating characteristic (ROC) curve (AUC), and predictive accuracy, as determined by calibration plots.

**Results:**

The incidence of AKI in our cohort was 19%. After initial LASSO variable selection, multivariate logistic regression revealed that age, pneumonia, D-dimer, and albumin were independently associated with AKI in hospitalized HF patients. The nomogram prediction model based on these independent predictors had AUCs of 0.760 and 0.744 in the training and test sets, respectively. The calibration plots indicate a strong concordance between the estimated AKI probabilities and the observed probabilities.

**Conclusions:**

A nomogram prediction model based on pneumonia, age, D-dimer, and albumin can help clinicians predict the risk of AKI in HF patients with moderate discriminability.

## 1. Introduction

Heart failure (HF) is the end stage and main cause of death in many cardiovascular disorders, seriously threatening the health of these patients. This condition affects 1 to 2% of adults and up to 70% of people over 70 [1]. In European countries, the median incidence of HF is 3.20 cases per 1000 people, and the median prevalence is 17.20 cases per 1000 people [2]. A common complication of acute heart failure is acute kidney injury (AKI), affecting 24%–45% of hospitalized patients with acute HF and acute kidney injury and occurring within one week of admission in 70%–90% of patients. Among HF patients, acute kidney injury can prolong the hospitalization time, worsen the prognosis, and increase the readmission rate and mortality [1]. Early detection and identification of renal deterioration can help to manage and prevent complications [3].

Many clinical factors may lead to AKI, but no one factor can help easily evaluate and predict AKI. Traditional medical statistics may ignore important factors due to the limitations of the methods, while extensive data analysis provides new insights. At the same time, there are racial differences in AKI [4]; however, there are few studies on heart failure patients in China.

To solve these difficulties, this study aimed to investigate the clinical characteristics and predictors of AKI in patients with HF. Least absolute shrinkage and selection operator (LASSO)–logistic regression was used to establish a clinical prediction model with moderate discriminability to predict the risk of AKI in hospitalized HF patients. This model helps reduce the incidence of adverse events during hospitalization.

## 2. Methods

### 2.1 Study Population and Protocol

Data for this study were obtained from the Anhui HF cohort study (a prospective, multicenter, observational clinical registry study), and the study design and findings have been described previously [5]. The patients included in the Anhui HF cohort were admitted to the Cardiology Department of the hospitals involved between December 2016 and October 2018 for acute HF or acute exacerbation of chronic HF. The inclusion criteria were: 1. Age >18 years; and 2. New York Heart Association (NYHA) functional classification II–IV (left ventricular ejection fraction (LVEF) ≤50% or LVEF >50% but N-terminal (NT)-pro-hormone brain natriuretic peptide (pro-BNP) >400 ng/L).

**Fig. 1.**
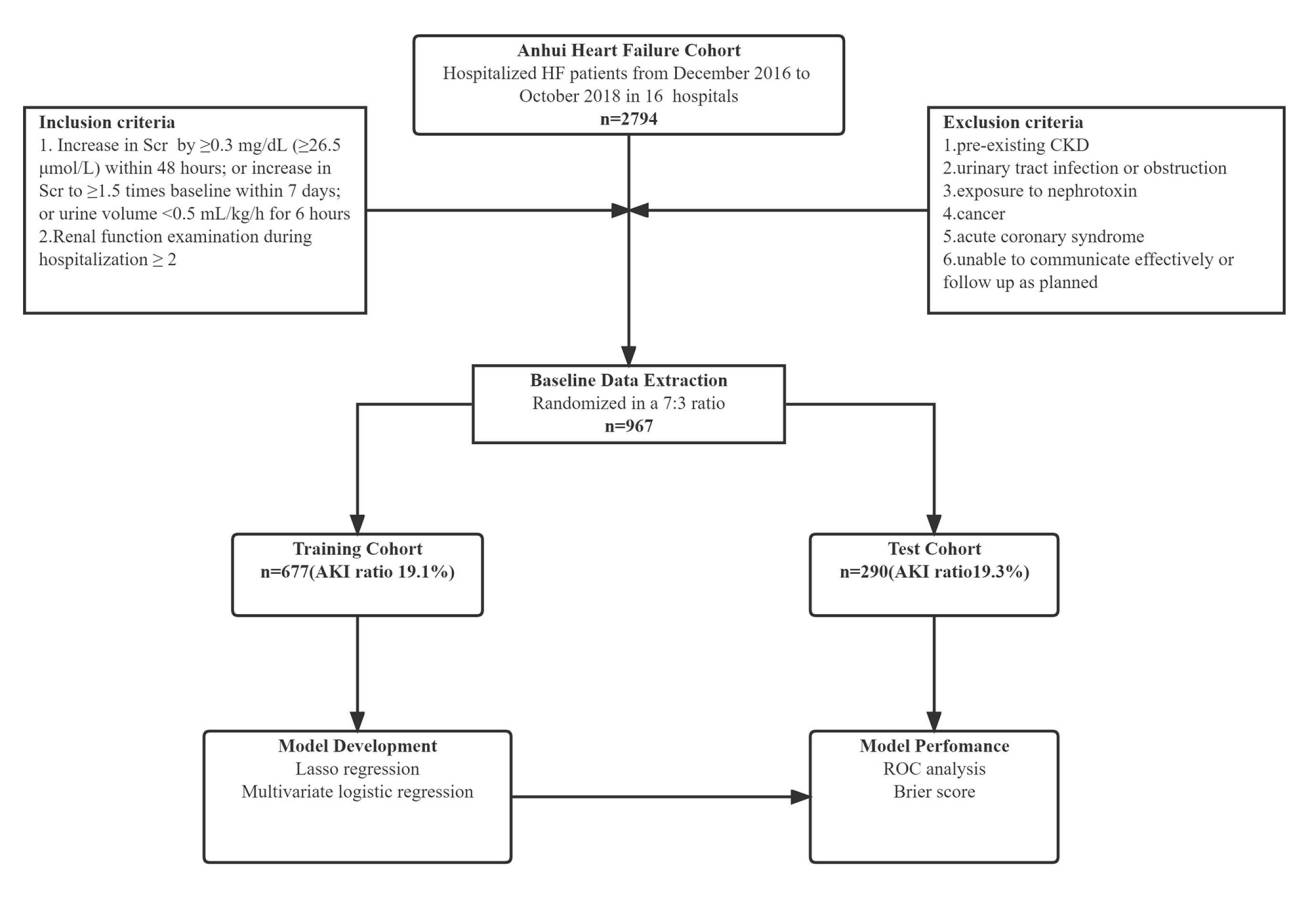
**Flow chart of the study**. CKD, chronic kidney disease; Scr, serum creatinine; AKI, acute kidney injury; ROC, receiver operating characteristic; HF, heart failure.

This study analyzed the baseline data of the Anhui HF cohort study, and the study endpoint was the occurrence of AKI events throughout hospitalization. AKI was defined according to the Kidney Disease: Improving Global Outcomes (KDIGO) Clinical Practice Guideline for Acute Kidney Injury [6] as an increase in serum creatinine (Scr) by ≥0.3 mg/dL (≥26.5 µmol/L) within 48 hours; an increase in Scr to ≥1.5 times the baseline that is known or presumed to have occurred within the prior 7 days; or urine volume <0.5 mL/kg/h for 6 hours. A detailed flow chart of the study protocol is shown in Fig. 1.

### 2.2 Data Collection and Preprocessing

Demographic data, medical history, cardiac surgery history, vital signs in the hospital, electrocardiography records during hospitalization, echocardiography records, laboratory test results, and drug treatment information were extracted from the patient’s baseline medical records and analyzed. Clinical variables (or features) with a ≥30% absence rate were deleted, and multiple interpolation was used for features with a deletion rate of <30%. Due to the large variation in the range of values for different clinical characteristics, Z score normalization was performed before data analysis. Finally, 98 clinical characteristics were employed in constructing the model (**Supplementary Table 1**).

### 2.3 Model Development and Evaluation

The subjects were randomly divided into the training or test set at a ratio of 7:3. Model development and preliminary evaluation were carried out in the training set, and model performance was verified in the test set. In the training set, LASSO regression was used to screen the variables. The value of log λ corresponding to the maximum area under the curve (AUC) was obtained by 10-fold cross-validation. The variables selected by LASSO regression were included in stepwise multivariate logistic regression, and variables whose *p* values < 0.05 were used to construct the predictive model. A nomogram was constructed from the model to visualize the risk of AKI in hospitalized HF patients. Receiver operating characteristic (ROC) curve analysis was performed on the training and test sets, and AUC was calculated to evaluate the discriminability of the predictive model. The calibration curves were drawn, and we utilized the Brier score to assess the calibration performance of our prediction model. Meanwhile, the performance of the model underwent internal validation by the bootstrap method (B = 1000) to reduce the risk of overfitting.

### 2.4 Statistical Analysis

Continuous variables are expressed as the mean and standard deviation or the median and interquartile range (IQR) based on the normality of variable distribution. Comparisons between groups of continuous variables were performed using the Student’s *t*-test (normal distribution) or Mann–Whitney U test. Categorical variables are expressed as frequencies (percentages), and the chi-square test or Fisher’s exact probability method was used to compare groups. A two-tailed *p* < 0.05 was defined as significantly different. Stata 17 software (Stata Corporation, College Station, TX, USA) was used for statistical analysis.

## 3. Results

A total of 2794 patients with heart failure were enrolled in the Anhui Heart Failure cohort from 16 participating hospitals. According to the inclusion and exclusion criteria, 967 patients were finally included. These patients were divided into the training set (677 patients, 19.1% with AKI) and the test set (290 patients, 19.3% with AKI). The mean age of the patients was 68.8 years, and 61.4% were male. The overall incidence of AKI during hospitalization was 19%. The main baseline characteristics of the patients are presented in Table 1 (See **Supplementary Table 2** for complete information).

**Table 1.** **Baseline characteristics of the hospitalized patients with heart failure**.

Characteristics		All (n = 967)	AKI (n = 185)	No AKI (n = 782)	*p*
Age (years)		68.8 ± 12.6	73.5 ± 12.0	67.7 ± 12.5	<0.001
Male, %		594 (61.4)	111 (60)	483 (61.8)	0.657
Body mass index (kg/m²)		24.0 ± 4.8	23.3 ± 4.3	24.1 ± 4.9	0.028
Systolic blood pressure (mmHg)		128.0 ± 20.7	128.8 ± 22.6	127.9 ± 20.2	0.581
Heart rate (bpm)		81 ± 18	84 ± 21	80 ± 17	0.006
CHD, n, %		476 (49.2)	86 (46.5)	390 (49.9)	0.407
COPD, n, %		62 (6.4)	10 (5.4)	52 (6.6)	0.534
Diabetes mellitus, n, %		210 (21.7)	48 (25.9)	162 (20.7)	0.121
Pneumonia, n, %		358 (37)	116 (63)	242 (31)	<0.001
Echocardiographic					
	LVEF (%)	45 (33, 60)	43 (32, 59)	45 (34, 61)	0.127
	FS (%)	24.6 ± 9.9	23.5 ± 9.4	24.9 ± 10.0	0.085
Laboratory examinations					
	NT–pro-BNP (ng/L)	2563 (1518, 4403)	3601 (2240, 5659)	2397 (1420, 3971)	<0.001
	RBC (10^9^/L)	4.1 ± 0.6	4.0 ± 0.7	4.1 ± 0.6	0.023
	BUN (mmol/L)	7.2 (5.6, 9.4)	7.8 (6.1, 10.6)	7.1 (5.5, 9.0)	0.001
	Albumin (g/L)	37.62 ± 5.58	35.8 ± 5.4	38.1 ± 5.5	<0.001
	UA (umol/L)	387 (304, 487)	422 (309, 524)	384 (303, 484)	0.094
	Cys C (mg/L)	1.17 (0.95, 1.51)	1.34 (1.03, 1.75)	1.14 (0.94, 1.43)	<0.001
	Sodium (mmol/L)	139.4 ± 4.2	138.5 ± 4.5	139.6 ± 4.1	0.002
	D-dimer (mg/L)	0.65 (0.39, 1.34)	1.18 (0.61, 2.37)	0.57 (0.36, 1.10)	<0.001
Medications					
	Loop diuretic, n, %	930 (96.2)	185 (100)	745 (95)	<0.001
	Beta blocker, n, %	645 (66.7)	102 (55)	543 (69)	<0.001
	Positive inotropic drugs, n, %	656 (67.8)	140 (75.7)	516 (66)	0.011
	Antibiotic, n, %	573 (59.3)	136 (73.5)	437 (55.9)	<0.001

CHD, coronary heart disease; COPD, chronic obstructive pulmonary disease; LVEF, left ventricular ejection fraction; FS, fraction shortening; NT-pro-BNP, N-terminal pro-B-type natriuretic peptide; RBC, red blood cell; BUN, blood urea nitrogen; UA, uric acid; Cys C, cystatin C; AKI, acute kidney injury.

**Fig. 2.**
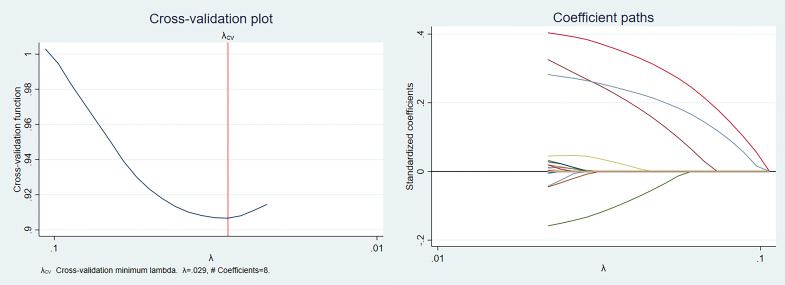
**The least absolute shrinkage and selection operator (LASSO) regression details**.

**Fig. 3.**
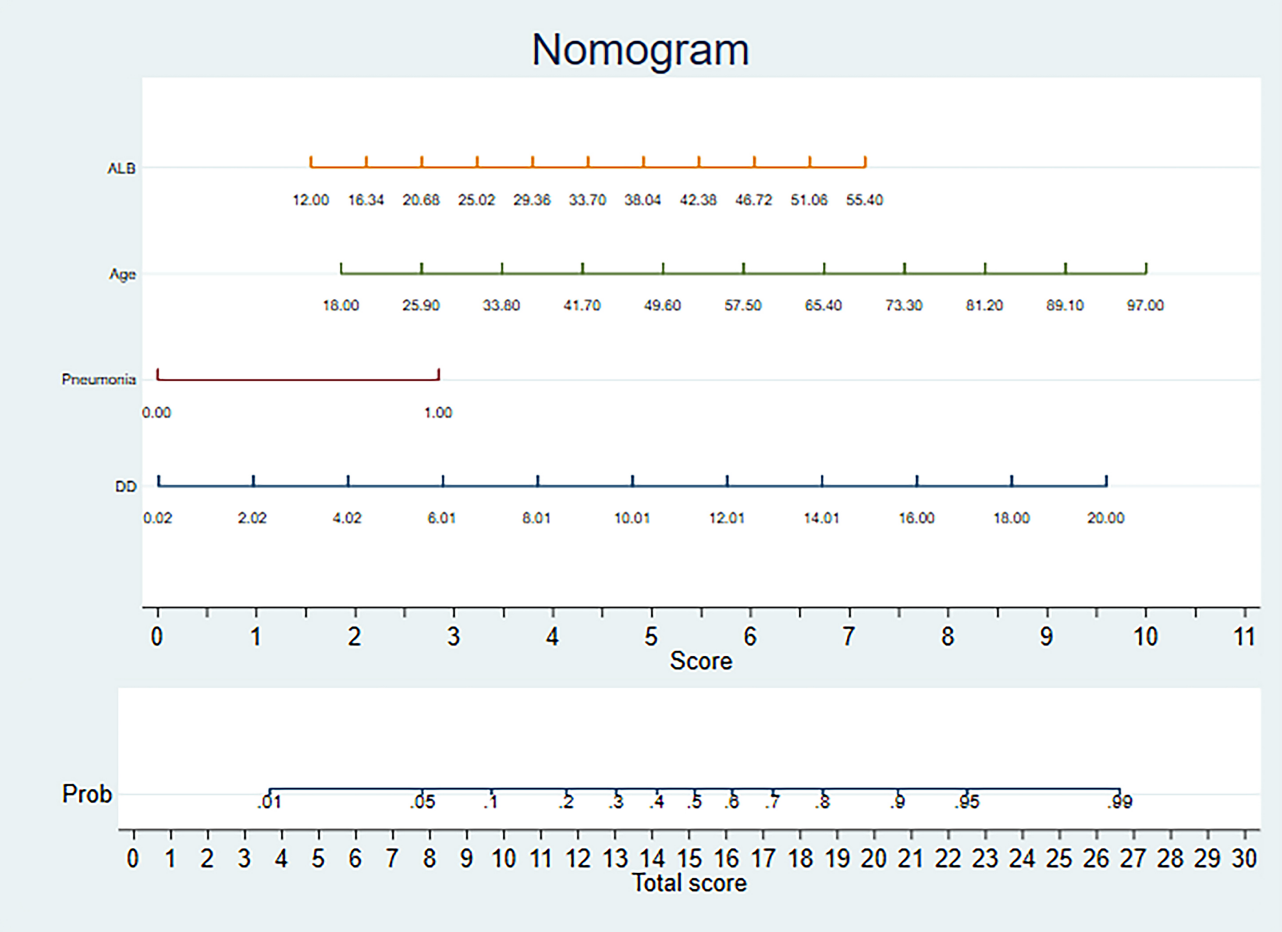
**Nomogram model for predicting the risk of AKI in hospitalized patients with heart failure**. Guidelines for using the Nomogram: Sketch a perpendicular line extending from the respective axis of each risk factor until it intersects the upper boundary labeled ‘POINTS’. Calculate the cumulative points for all risk factors and draw a descending line from the axis marked ‘TOTAL POINTS’ until it intersects the risk axes to determine AKI probability for hospitalized patients with heart failure. For binary variables, 0 = no and 1 = yes. AKI, acute kidney injury; ALB, albumin; DD, D-dimer.

**Fig. 4.**
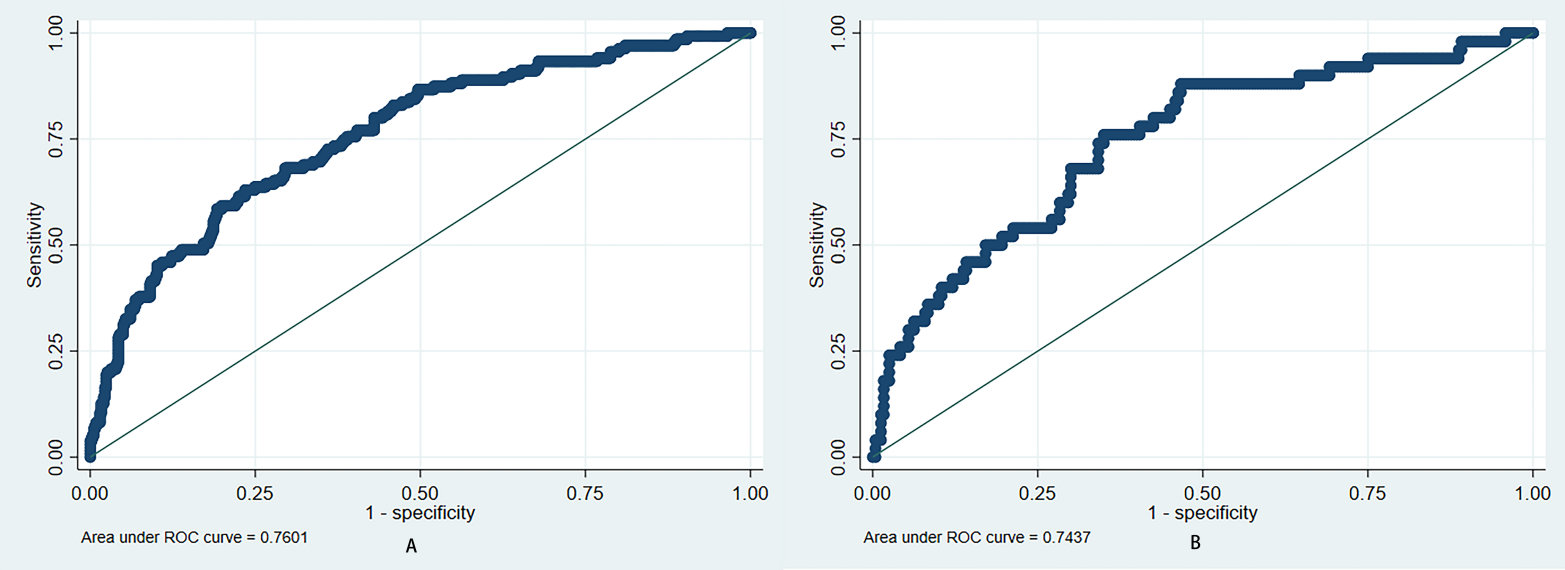
**Discrimination of prediction model**. (A) Training set: The AUC of the ROC curve was 0.760 (95% CI: 0.714–0.806). (B) Test set: The AUC of the ROC curve was 0.744 (95% CI: 0.667–0.82). AUC, area under the curve; ROC, receiver operating characteristic; CI, confidence interval.

**Fig. 5.**
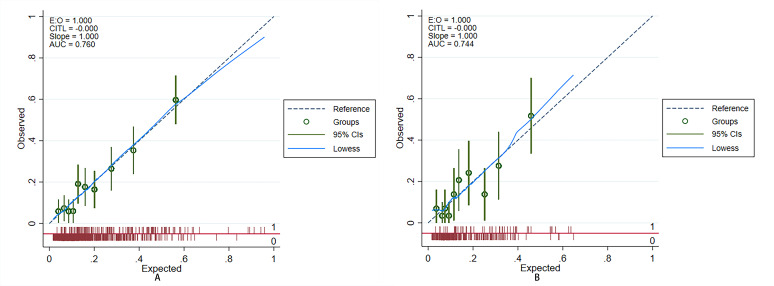
**Calibration of prediction model**. (A) Training set: The Brier score for the model was 0.161. (B) Test set: The Brier score for the model was 0.123. AUC, area under the curve; CIs, confidence intervals; E:O, expected versus observed ratios; CITL, calibration-in-the-large.

**Fig. 6.**
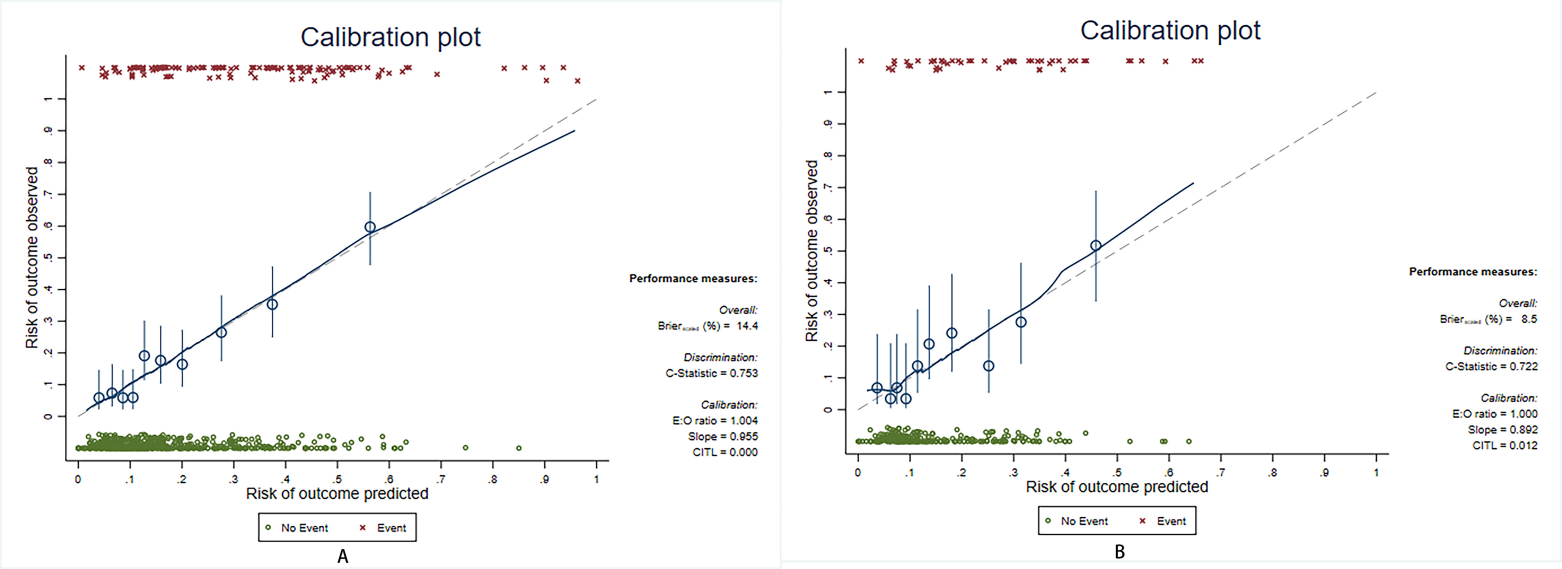
**Bootstrap method results**. (A) Training set: The AUC of the ROC curve was 0.753 (95% CI 0.709–0.799), and the Brier score for the model was 0.144. (B) Test set: The AUC of the ROC curve was 0.722 (95% CI 0.648–0.799), and the Brier score for the model was 0.085. AUC, area under the curve; CI, confidence interval; E:O, expected versus observed ratios; CITL, calibration-in-the-large; ROC, receiver operating characteristic.

Eight potential AKI predictors were selected by LASSO regression: Pneumonia, age, D-dimer, brain natriuretic peptide (BNP), albumin, hematocrit, serum creatine kinase, and beta blockers. The details of the LASSO regression are shown in Fig. 2. After multivariate logistic regression, age [OR 1.04 (1.02, 1.06), *p* < 0.001], pneumonia [OR 2.97 (1.93, 4.57), *p* < 0.001], D-dimer [OR 1.19 (1.09, 1.31), *p* < 0.001], and albumin [OR 0.95 (0.91, 0.99), *p* = 0.009] were identified as independent predictors of an AKI event in hospitalized HF patients (Table 2). To make the prediction model more suitable for use in the clinic, the four independent predictors were used for the development of a visual scoring system for the risk of AKI in hospitalized HF patients Fig. 3. In the training and test sets, the AUCs of the prediction model were 0.760 (95% CI: 0.714–0.806) and 0.744 (95% CI: 0.667–0.82), and the Brier scores were 0.161 and 0.123, respectively. The results of the bootstrap method showed that the prediction model had moderate discriminability (AUC: 0.753 (95% CI 0.709–0.799) in the training set and 0.722 (95% CI 0.648–0.799) in the test set) and good calibration (Brier score: 0.144 in the training set and 0.085 in the test set). The ROC and calibration curves of the prediction model are shown in Figs. 4,5, respectively. The results of the bootstrap method are shown in Fig. 6.

**Table 2.** **Independent risk factors for AKI in hospitalized patients with heart failure after multivariate regression**.

	Z	OR with CI	*p*
Pneumonia	4.95	2.971 (1.930~4.572)	<0.001
Age (years)	4.37	1.043 (1.023~1.623)	<0.001
D-dimer (mg/L)	3.70	1.193 (1.086~1.309)	<0.001
Albumin (g/L)	–2.62	0.947 (0.910~0.986)	0.009

OR, odds ratio; CI, confidence interval; AKI, acute kidney injury.

## 4. Discussion

This study aimed to identify independent predictors of AKI in hospitalized HF patients and validate a nomogram risk prediction model established with those factors. In this study, we identified four independent risk factors that can predict AKI in hospitalized HF patients: Pneumonia, age, D-dimer, and albumin. Based on these independent risk factors, we developed a nomogram prediction model to predict the risk of AKI in hospitalized HF patients. The AUC for the predictive nomogram was 0.760 in the training set and 0.744 in the test set. The calibration plots indicate a strong concordance between the estimated AKI probabilities and the observed probabilities.

Although the mechanisms of acute kidney injury in individuals experiencing acute heart failure are known to mainly involve hemodynamic and non-hemodynamic factors [7], the exact underlying mechanism remains unclear. Therefore, we have more reasons to conduct further research on this topic in the context of extensive data analysis.

Advanced age is known to be a common risk factor for AKI, as demonstrated in several clinical studies [8, 9]. Although AKI occurs at all ages, its relevance is higher in intensive and critical medicine in the older population, and the mortality rate may be 50% or higher [10]. The probability of AKI in the elderly population is 3–55 times higher than that in the young [11, 12], and the incidence increased significantly from 2008 to 2018 [13]. Increased susceptibility to AKI in older patients may be associated with decreased renal function, altered renal vascular reactivity, multiple drug use, and comorbidities [14]. In response, Neil G Docherty *et al*. [15] proposed that hemodynamic frailty, such as the state of frailty in older patients, is a state of increased disease susceptibility prevalent in older adults, characterized by impaired compensatory response networks that control circulatory capacity and adaptive hemodynamic function. An important reason relating to why older patients are prone to hemodynamic weakness is the impaired ability to maintain systemic water balance. Dehydration and decreased circulating blood volume may lead to insufficient tissue perfusion and reduce renal blood flow, thus causing AKI, making it an important factor for the increased risk of AKI [15].

Pulmonary inflammation affects renal function through hemodynamic, neurohormonal, proinflammatory, and proapoptotic effects [16], and there is also extensive cross-linking between lung diseases [17]. Lung and kidney diseases reinforce and aggravate each other, creating a vicious cycle [17]. Lung disease can cause kidney damage through a variety of pathways, including inflammation/immune-mediated damage, hypoxemia, hypercapnia, and nephrotoxic substances. After AKI, factors such as increased release of inflammatory mediators, excess body fluids, and increased risk of infection caused by immune dysfunction can further aggravate the severity of lung disease and lead to respiratory failure. In previous studies, AKI itself was identified as a common complication of community-acquired pneumonia (CAP) [18], with its incidence possibly being due to the severity of the pneumonia [19]. When there is an increase in inflammatory stress, it can potentially contribute to the development of cardiovascular disease (CVD). Inflammation is both a cause and an aggravating factor in CVD, as well as a mediator of its worst prognosis [20].

Sodium–glucose cotransporter 2 (SGLT2), predominantly expressed in the proximal renal tubules, plays a pivotal role in glucose transport across the epithelium. Emerging evidence suggests that it is upregulated in cardiomyocytes of patients suffering from heart failure [21], and its potential proinflammatory activity may impact cardiovascular function through excessive inflammation [22, 23]. The cardio–renal protective mechanism of SGLT2 inhibitors (SGLT2i) may be attributed to their ability to ameliorate inflammation and oxidative stress [24, 25], which is consistent with our study’s observation that inflammation could contribute to AKI development.

The D-dimer level positively correlates with inflammatory indicator levels, such as the C-reactive protein (CRP) [26]. There are extensive interactions between inflammatory reactions and coagulation activation [27]. Therefore, increased D-dimer levels may induce AKI through non-hemodynamic mechanisms such as inflammation [28]. Venous thrombosis is common in kidney diseases, although its mechanism remains unclear. Dekkers *et al*. [29] believe that renal dysfunction and vascular impairment are related to increased coagulation-promoting factors. At the same time, renal and vascular functions are significant factors that contribute to developing venous thrombosis. [29]. This study suggests that the detected hypercoagulability may be the manifestation of impaired renal function. Recent studies have consistently supported our finding that an elevated D-dimer level is an independent predictor of AKI [30, 31]. In conclusion, the increase in the D-dimer level can not only promote the deterioration of renal function but also may be one of the indicators of renal function impairment.

Hypoalbuminemia is a recognized risk factor for AKI [32]. Albumin plays a crucial part in the physiological functions of macromolecules and carriers *in vivo*, and a decrease in serum albumin may lead to multiple organ dysfunction. Low serum albumin levels indicate inflammation severity [33] and may induce AKI through non-hemodynamic mechanisms. Similar to previous studies [34], our study found that hypoalbuminemia is a risk factor for AKI. A recent meta-analysis also demonstrated that hypoproteinemia has been linked to a higher likelihood of AKI [35]. Shao *et al*. [36] also suggested that serum albumin can protect renal function through various mechanisms.

Compared with previous studies on AKI in hospitalized patients with HF in China, our research has made more progress [8, 9]. Most previous studies used traditional medical statistics methods to identify independent risk factors for AKI, but AKI is a clinical syndrome with complex mechanisms; therefore, these traditional methods may be unable to identify a number of independent risk factors. For example, the study by Hu *et al*. [34] did not include echocardiography data in their analyses. In this study, LASSO regression was used for variable selection to solve potential multicollinearity problems among multiple variables, allowing the selection of 11 possible influencing factors from a total of 98 factors. Then, following multivariate logistic regression analysis, six independent predictors of AKI were finally identified in HF patients during hospitalization. These factors were used to create a prediction model that demonstrated a moderate degree of discriminability and calibration. Finally, it was transformed into a nomogram, which is more convenient for clinical use. Additionally, the data in this study came from a regional, multicenter, prospective heart failure cohort with a large sample size and good representativeness, which can provide support for clinical practice.

However, our study has some limitations. First, our study did not identify novel biomarkers, such as kidney injury molecule (KIM)-1 levels. More markers related to heart failure and kidney injury can be detected in the future, making the prediction model more accurate. Second, although our research is a multicenter study, it is limited to Anhui province, meaning the generalizability of the nomogram prediction model for other people remains to be verified. This study only conducted internal validation of the study population, and further external validation can be conducted in more clinical patients in the future to discuss its applicability. Finally, we did not evaluate the estimated glomerular filtration rate (eGFR) of the selected participants to determine whether they were complicated with chronic kidney disease, which may have affected the results. In the future, eGFR can be rigorously calculated to prepare for future prospective studies.

## 5. Conclusions

In conclusion, pneumonia, age, D-dimer, and albumin were independent predictors of AKI in hospitalized HF patients. A nomogram predictive model utilizing these four variables has the potential to assist clinicians in accurately estimating the likelihood of AKI occurrence among hospitalized HF patients. It is helpful to reduce the occurrence of adverse events in patients with heart failure during hospitalization.

## Data Availability

Our manuscript has data associated with it in a data repository.

## Supplementary Material

Supplementary material associated with this article can be found, in the online version, at https://doi.org/10.31083/j.rcm2508293.

## References

[1] Ismail Y, Kasmikha Z, Green HL, McCullough PA (2012). Cardio-renal syndrome type 1: epidemiology, pathophysiology, and treatment. *Seminars in Nephrology*.

[2] Seferović PM, Vardas P, Jankowska EA, Maggioni AP, Timmis A, Milinković I (2021). The Heart Failure Association Atlas: Heart Failure Epidemiology and Management Statistics 2019. *European Journal of Heart Failure*.

[3] Mostafa A, Said K, Ammar W, Eltawil AE, Abdelhamid M (2020). New renal haemodynamic indices can predict worsening of renal function in acute decompensated heart failure. *ESC Heart Failure*.

[4] Lunyera J, Clare RM, Chiswell K, Scialla JJ, Pun PH, Thomas KL (2021). Racial Differences in AKI Incidence Following Percutaneous Coronary Intervention. *Journal of the American Society of Nephrology: JASN*.

[5] Wang Q, Li B, Chen K, Yu F, Su H, Hu K (2021). Machine learning-based risk prediction of malignant arrhythmia in hospitalized patients with heart failure. *ESC Heart Failure*.

[6] Kellum JA, Lameire N, Aspelin P, Barsoum RS, Burdmann EA, Goldstein SL (2012). Kidney disease: improving global outcomes (KDIGO) acute kidney injury work group. KDIGO clinical practice guideline for acute kidney injury. *Kidney International Supplements*.

[7] Virzì GM, Clementi A, Brocca A, de Cal M, Vescovo G, Granata A (2014). The hemodynamic and nonhemodynamic crosstalk in cardiorenal syndrome type 1. *Cardiorenal Medicine*.

[8] Fan Z, Li Y, Ji H, Jian X (2018). Nomogram Model to Predict Cardiorenal Syndrome Type 1 in Patients with Acute Heart Failure. *Kidney & Blood Pressure Research*.

[9] Zhou LZ, Yang XB, Guan Y, Xu X, Tan MT, Hou FF (2016). Development and Validation of a Risk Score for Prediction of Acute Kidney Injury in Patients With Acute Decompensated Heart Failure: A Prospective Cohort Study in China. *Journal of the American Heart Association*.

[10] Hoste EAJ, Bagshaw SM, Bellomo R, Cely CM, Colman R, Cruz DN (2015). Epidemiology of acute kidney injury in critically ill patients: the multinational AKI-EPI study. *Intensive Care Medicine*.

[11] Ali T, Khan I, Simpson W, Prescott G, Townend J, Smith W (2007). Incidence and outcomes in acute kidney injury: a comprehensive population-based study. *Journal of the American Society of Nephrology: JASN*.

[12] Feest TG, Round A, Hamad S (1993). Incidence of severe acute renal failure in adults: results of a community based study. *BMJ (Clinical Research Ed.)*.

[13] Khan S, Loi V, Rosner MH (2017). Drug-Induced Kidney Injury in the Elderly. *Drugs & Aging*.

[14] Yokota LG, Sampaio BM, Rocha EP, Balbi AL, Sousa Prado IR, Ponce D (2018). Acute kidney injury in elderly patients: narrative review on incidence, risk factors, and mortality. *International Journal of Nephrology and Renovascular Disease*.

[15] Docherty NG, Delles C, D’Haese P, Layton AT, Martínez-Salgado C, Vervaet BA (2021). Haemodynamic frailty - A risk factor for acute kidney injury in the elderly. *Ageing Research Reviews*.

[16] Husain-Syed F, Slutsky AS, Ronco C (2016). Lung-Kidney Cross-Talk in the Critically Ill Patient. *American Journal of Respiratory and Critical Care Medicine*.

[17] Joannidis M, Forni LG, Klein SJ, Honore PM, Kashani K, Ostermann M (2020). Lung-kidney interactions in critically ill patients: consensus report of the Acute Disease Quality Initiative (ADQI) 21 Workgroup. *Intensive Care Medicine*.

[18] Chen D, Yuan H, Cao C, Liu Z, Jiang L, Tan Y (2021). Impact of acute kidney injury on in-hospital outcomes in Chinese patients with community acquired pneumonia. *BMC Pulmonary Medicine*.

[19] Akram AR, Singanayagam A, Choudhury G, Mandal P, Chalmers JD, Hill AT (2010). Incidence and prognostic implications of acute kidney injury on admission in patients with community-acquired pneumonia. *Chest*.

[20] Sardu C, Paolisso G, Marfella R (2020). Inflammatory Related Cardiovascular Diseases: From Molecular Mechanisms to Therapeutic Targets. *Current Pharmaceutical Design*.

[21] Marfella R, Scisciola L, D’Onofrio N, Maiello C, Trotta MC, Sardu C (2022). Sodium-glucose cotransporter-2 (SGLT2) expression in diabetic and non-diabetic failing human cardiomyocytes. *Pharmacological Research*.

[22] Sardu C, Gatta G, Pieretti G, Onofrio ND, Balestrieri ML, Scisciola L (2023). SGLT2 breast expression could affect the cardiovascular performance in pre-menopausal women with fatty vs. non fatty breast via over-inflammation and sirtuins’ down regulation. *European Journal of Internal Medicine*.

[23] D’Onofrio N, Sardu C, Trotta MC, Scisciola L, Turriziani F, Ferraraccio F (2021). Sodium-glucose co-transporter2 expression and inflammatory activity in diabetic atherosclerotic plaques: Effects of sodium-glucose co-transporter2 inhibitor treatment. *Molecular Metabolism*.

[24] Salvatore T, Galiero R, Caturano A, Rinaldi L, Di Martino A, Albanese G (2022). An Overview of the Cardiorenal Protective Mechanisms of SGLT2 Inhibitors. *International Journal of Molecular Sciences*.

[25] Paolisso P, Bergamaschi L, Santulli G, Gallinoro E, Cesaro A, Gragnano F (2022). Infarct size, inflammatory burden, and admission hyperglycemia in diabetic patients with acute myocardial infarction treated with SGLT2-inhibitors: a multicenter international registry. *Cardiovascular Diabetology*.

[26] Lu J, Wang X, Chen Q, Chen M, Cheng L, Jiang H (2016). D-dimer Is a Predictor of 28-Day Mortality in Critically Ill Patients Receiving Continuous Renal Replacement Therapy. *Archives of Medical Research*.

[27] Iba T, Levy JH (2018). Inflammation and thrombosis: roles of neutrophils, platelets and endothelial cells and their interactions in thrombus formation during sepsis. *Journal of Thrombosis and Haemostasis: JTH*.

[28] Ronco C, Bellasi A, Di Lullo L (2019). Implication of Acute Kidney Injury in Heart Failure. *Heart Failure Clinics*.

[29] Dekkers IA, de Mutsert R, de Vries APJ, Rosendaal FR, Cannegieter SC, Jukema JW (2018). Determinants of impaired renal and vascular function are associated with elevated levels of procoagulant factors in the general population. *Journal of Thrombosis and Haemostasis: JTH*.

[30] Wang B, Jiang Q, Wu X (2020). Association of D-dimers with acute kidney injury in pregnant women: a retrospective study. *The Journal of International Medical Research*.

[31] Yildirim C, Ozger HS, Yasar E, Tombul N, Gulbahar O, Yildiz M (2021). Early predictors of acute kidney injury in COVID-19 patients. *Nephrology (Carlton, Vic.)*.

[32] Hoste EAJ, Kellum JA, Selby NM, Zarbock A, Palevsky PM, Bagshaw SM (2018). Global epidemiology and outcomes of acute kidney injury. *Nature Reviews. Nephrology*.

[33] Soeters PB, Wolfe RR, Shenkin A (2019). Hypoalbuminemia: Pathogenesis and Clinical Significance. *JPEN. Journal of Parenteral and Enteral Nutrition*.

[34] Hu W, He W, Liu W, Fang X, Wu Y, Yu F (2016). Risk Factors and Prognosis of Cardiorenal Syndrome Type 1 in Elderly Chinese Patients: A Retrospective Observational Cohort Study. *Kidney & Blood Pressure Research*.

[35] Hansrivijit P, Yarlagadda K, Cheungpasitporn W, Thongprayoon C, Ghahramani N (2021). Hypoalbuminemia is associated with increased risk of acute kidney injury in hospitalized patients: A meta-analysis. *Journal of Critical Care*.

[36] Shao M, Wang S, Parameswaran PK (2017). Hypoalbuminemia: a risk factor for acute kidney injury development and progression to chronic kidney disease in critically ill patients. *International Urology and Nephrology*.

