# Supplementary material for: Nomogram Model to Predict Acute Kidney Injury in Hospitalized Patients with Heart Failure
Source: Rev Cardiovasc Med. 2024 Aug 20;25(8):293. doi: 10.31083/j.rcm2508293 (PMC11367008; doi:10.31083/j.rcm2508293)
Supplement: Supplementary file 1 [file 2153-8174-25-8-293-s1.docx]

**Supplementary Material**

**Supplementary Table 1. Clinical features used for model development.**

**Supplementary Table 2. Baseline characteristics of patients with and without acute kidney injury (AKI).**

**Supplementary Table 1.** Clinical features used for model development

| **Characteristics** | **Echocardiography** | **Laboratory examinations** | **Medications and device therapies** | |
| --- | --- | --- | --- | --- |
| Age  Male  Body mass index  Temperature  Heart rate  Respiratory rate  Systolic blood pressure  Diastolic blood pressure  CHD  Hypertension  Diabetes mellitus  COPD  Pneumonia  AF  Thyroid disease | LA  LVEDD  LVESD  LVEF  FS  TRPG  SPAP | Routine blood tests:  WBC, Neutrophil, Lymphocyte, Monocyte, Eosinophilic, Basophil, RBC,  HGB, HCT, MCV,  MCHC, PLT, MPV, PCT, Ret  Blood biochemical tests：  ALP, ALT, AST, BUN, TBIL, DBIL, IBIL,  PAB, TP, Albumin, GLB, UA,  Cys C, CO2, GLU, phosphorus, potassium, sodium, calcium, magnesium, chlorine, AGP, OSM, TC, TG, HDLC, LDLC, VLDLC.  Blood coagulation tests:  PT, INR, APTT, TT, D-dimer, FIB.  Thyroid hormones:  FT3, FT4, TSH.  Others:  NT-proBNP, MYO, LDH, CK, CKMB.  urine SG, urine PH | Ablation  CRT/ICD  PCI/CABG  ACEI/ARB  Beta Blocker  Antisterone  CCB  Anticoagulant  Antiplatelet  Statin  Loop diuretic  Oral anti-arrhythmic Antibiotic  Positive inotropic drugs  Venous dilatation  Nutritional myocardium drugs  CN medicine |  |

**Abbreviations** CHD: coronary heart disease; COPD: chronic obstructive pulmonary disease; AF: Atrial fibrillation; NT-pro BNP: N-terminal pro-B-type natriuretic peptide; RBC: red blood cell; WBC: white blood cell; PLT: platelets; HGB: hemoglobin; HCT: hematocrit; MCV: mean corpuscular volume; MCHC: mean corpuscular hemoglobin contentration; MPV: mean platelet volume; BUN: blood urea nitrogen; Ret: reticulocyte; ALP: alkaline phosphatase; ALT: alanine aminotransferase; AST: aspartate aminotransferase; PCT: thrombocytocrit; TBIL: total bilirubin; DBIL: direct bilirubin; IBIL: indirect bilirubin; PAB: prealbumin；TP: total protein; GLB: globulin; Scr: serum creatinine；UA: uric acid; Cys C: cystatin C; GLU: serum glucose; AGP: anionic gap; OSM: osmotic pressure; MYO: myoglobin; LDH: lactate dehydrogenase; CKMB: creatine phosphokinase isoenzyme; CK : creatine phosphokinase; PT: prothrombin time; INR: international standardized ratio; APTT: activated partial thromboplastin time; TT: thrombin time; FIB: fibrinogen; TC: serum total cholesterol; TG: triglyceride; LDLC: low density lipoprotein cholesterin; HDLC: high density lipoprotein cholesterin; VLDLC: very low density lipoprotein cholesterin;FT3:free triiodothyronine;FT4:free thyroxine; TSH: thyroid-stimulating hormone; ACEI: Angiotensin converting enzyme inhibitor ;ARB: angiotensin receptor blocker; CCB: calcium channel blockers; SG: specific gravity ; PCI: Percutaneous coronary intervention; CABG: coronary artery bypass grafting: CRT: cardiac resynchronization therapy; ICD: implantable cardioverter defibrillator: LA: left atrium LVEF: left ventricular ejection fraction; FS: fractional shortening; left ventricular end diastolic dimension; LVESD: left ventricular end systolic dimension; SPAP: systolic pulmonary artery pressure; TRPG: tricuspid regurgitation peak gradient.

**Supplementary Table 2.** Baseline characteristics of patients with and without acute kidney injury (AKI).

|  | ALL（n=967） | AKI（n=185） | No AKI （n=782） | P |
| --- | --- | --- | --- | --- |
| **Characteristics** |  |  |  |  |
| Age (years) | 68.8±12.6 | 73.5±12.0 | 67.7±12.5 | ＜0.001 |
| Male,% | 594(61.4) | 111(60) | 483(61.8) | 0.657 |
| Body mass index(kg/m²) | 24.0±4.8 | 23.3±4.3 | 24.1±4.9 | 0.028 |
| Systolic blood pressure (mmHg) | 128.0±20.7 | 128.8±22.6 | 127.9±20.2 | 0.581 |
| Diastolic blood pressure (mmHg) | 78.6±13.4 | 79.5±14.5 | 78.1±13.1 | 0.201 |
| Heart rate (bpm) | 81±18 | 84±21 | 80±17 | 0.006 |
| Respiratory rate (cycle peminute)，n | 19.2±1.05 | 19.4±1.3 | 19.2±1.0 | 0.125 |
| Temperature (℃) | 36.4±0.3 | 36.3±0.4 | 36.4±0.3 | 0.203 |
| **Medical history** |  |  |  |  |
| CHD，n，% | 476 (49.2) | 86(46.5) | 390(49.9) | 0.407 |
| Hypertension，n，% | 488(50.5) | 96(51.9) | 392(50.1) | 0.666 |
| Diabetes mellitus,，n，% | 210(21.7) | 48(25.9) | 162(20.7) | 0.121 |
| Pneumonia，n，% | 358(37) | 116(63) | 242(31) | ＜0.001 |
| COPD，n，%  AF，n，%  Thyroid disease，n，% | 62(6.4)  351(36.3)  36(3.7) | 10(5.4)  73(39.5)  5(2.7) | 52(6.6)  278(35.5)  31(4) | 0.534  0.32  0.415 |
| **Laboratory examinations** |  |  |  |  |
| NT-pro BNP(ng/L) | 2563(1518,4403) | 3601(2240,5659) | 2397(1420,3971) | ＜0.001 |
| WBC (10^9/L) | 6.2(5.0,7.9) | 6.5（5.0,8.4) | 6.1(5.0,7.7) | 0.147 |
| RBC (10^9/L) | 4.1±0.6 | 4.0±0.7 | 4.1±0.6 | 0.023 |
| PLT(10^9/L) | 162(127,203) | 154(120，190) | 164(128，206) | 0.045 |
| HGB(g/L) | 124±19 | 122±21 | 125±19 | 0.077 |
| Neutrophil(10^9/L) | 4.0(3.2,5.4) | 4.3(3.3,6.5) | 4.0(3.1,5.2) | 0.01 |
| Monocyte | 0.48(0.37,0.63) | 0.50(0.36,0.66) | 0.48(0.37,0.63) | 0.334 |
| Lymphocyte(10^9/L) | 1.3(1.0,1.8) | 1.2(0.7，1.6) | 1.4(1.0,1.8) | ＜0.001 |
| Basophil | 0.03(0.02,0.04) | 0.03(0.02,0.04) | 0.03(0.02,0.04) | 0.858 |
| Eosinophilic(10^9/L) | 0.08(0.04,0.14) | 0.05(0.02,0.12) | 0.08(0.04,0.15) | ＜0.001 |
| HCT(L/L) | 0.38(0.34,041) | 0.38(0.34,0.41) | 0.38(0.34,0.42) | 0.131 |
| MCV(fl) | 92.3±6.1 | 92.8±5.7 | 2±6.2 | 0.191 |
| MCHC(pg) | 329.0±9.9 | 328.1±9.5 | 329.2±10.0 | 0.138 |
| MPV(fl) | 10.3±1.8 | 10.2±1.8 | 10.4±1.8 | 0.197 |
| PCT(%) | 0.16(0.13，0.20) | 0.15(0.12,0.20) | 0.17(0.13,0.20) | 0.007 |
| Ret(10^12/L) | 0.07(0.05,0.09) | 0.07(0.05,0.09) | 0.07(0.05,0.09) | 0.795 |
| ALP(IU/L) | 72(59,90) | 72(60,90) | 73(59,90) | 0.821 |
| ALT(IU/L) | 22(15,36) | 20(13,36) | 22(15,36) | 0.160 |
| AST(IU/L) | 25(19,37) | 25(20,42) | 25(19,36) | 0.607 |
| BUN(mmol/L) | 7.2(5.6,9.4) | 7.8(6.1,10.6) | 7.1(5.5,9.0) | 0.001 |
| TBIL(umol/L) | 15.3(10.5，23.6) | 17.4(11.6,26.9) | 14.8(10.2,22.8) | 0.003 |
| DBIL(umol/L) | 5.3(3.7,8.2) | 5.9(4.2，9.1) | 5.2(3.6,7.7) | 0.005 |
| IBIL(umol/L) | 9.7(6.5,15.5) | 11.1(7.3,17.1) | 9.4(6.4,15.0) | 0.026 |
| PAB(mg/L) | 213±72 | 192±76 | 218±71 | ＜0.001 |
| TP(g/L) | 63.7±7.4 | 62.4±7.6 | 64.0±7.4 | 0.006 |
| Albumin (g/L) | 37.62±5.58 | 35.8±5.4 | 38.1±5.5 | ＜0.001 |
| GLB(g/L) | 26.1±5.5 | 26.6±6.1 | 25.9±6.4 | 0.149 |
| UA(umol/Ll) | 387(304,487) | 422(309，524) | 384(303,484) | 0.094 |
| Cys C(mg/L) | 1.17(0.95,1.51) | 1.34(1.03,1.75) | 1.14(0.94,1.43) | ＜0.001 |
| CO2(mmol/L) | 22.8±3.6 | 24.7±4.2 | 24.9±3.5 | 0.47 |
| phosphorus (mmol/L) | 1.19±0.23 | 1.18±0.25 | 1.19±0.23 | 0.46 |
| GLU(mmol/L) | 7.62±3.24 | 7.65±3.22 | 7.62±3.25 | 0.885 |
| potassium (mmol/L) | 3.96±0.52 | 3.96±0.59 | 3.96±0.50 | 0.905 |
| sodium (mmol/L) | 139.4±4.2 | 138.5±4.5 | 139.6±4.1 | 0.002 |
| chlorine (mmol/L) | 103.6±5.3 | 102.6±5.8 | 103.8±5.2 | 0.01 |
| calcium (mmol/L) | 2.23±0.17 | 2.20±0.18 | 2.24±0.17 | 0.011 |
| magnesium (mmol/L) | 0.82±0.12 | 0.84±0.15 | 0.82±0.12 | 0.205 |
| AGP(mmol/L) | 10.8±3.7 | 11.1±3.8 | 10.8±3.7 | 0.257 |
| OSM(mOsm/L) | 288.7±8.0 | 283.0±9.3 | 283.8±7.7 | 0.257 |
| MYO(g/L) | 24(16,41) | 32(17,65) | 22.5(15,38) | ＜0.001 |
| LDH(IU/L) | 200(162,276) | 214(166,308) | 196(161,267) | 0.039 |
| CKMB(IU/L) | 16(12,20) | 16(12,21) | 15(12,20) | 0.169 |
| CK(IU/L) | 70(48,104) | 72(48,116) | 70(48,102) | 0.668 |
| PT(s) | 13.7(12.8,15.1) | 14(13.1,15.5) | 13.7(12.8,15.0) | 0.023 |
| INR | 1.1(1.0,1.2) | 1.1(1.0,1.2) | 1.1(1.0,1.2) | 0.013 |
| APTT(s) | 35.9(32.3，40.7) | 36.6(32.5,41.9) | 35.7(32.3,40.5) | 0.127 |
| TT(s) | 17.1(16.2，18.2) | 17.1(16.2，18.2) | 17.1(16.2，18.2) | 0.936 |
| D-dimer (mg/L) | 0.65(0.39,1.34) | 1.18(0.61,2.37) | 0.57(0.36，1.10) | ＜0.001 |
| FIB(g/L) | 3.3(2.8,4.0) | 3.3(2.7,4.2) | 3.3(2.7,4.0) | 0.318 |
| TC(mmol/L) | 4.06±1.10 | 4.08±1.20 | 4.06±1.08 | 0.878 |
| TG(mmol/L) | 1.15(0.88，1.56) | 1.09(0.85，1.42) | 1.17(0.88，1.57) | 0.038 |
| LDLC(mmol/L) | 2.05(1.59,2.55) | 2.03(1.59,2.60) | 2.05(1.59,2.55) | 0.818 |
| HDLC(mmol/L) | 0.96(0.77，1.18) | 0.98(0.79，1.18) | 0.95(0.77，1.18) | 0.796 |
| VLDLC(mmol/L) | 0.89(0.73，1.09) | 0.90(0.71，1.09) | 0.89(0.74，1.10) | 0.722 |
| FT3(pmol/L) | 4.5±1.2 | 4.2±1.0 | 4.5±1.3 | 0.001 |
| FT4(pmol/L) | 15.7±3.9 | 15.8±3.4 | 15.0±4.0 | 0.009 |
| TSH(mIU/L) | 2.15(1.26,3.50) | 1.95(1.12,3.54) | 2.21(1.29,3.48) | 0.224 |
| urine SG | 1.02±0.01 | 1.02±0.01 | 1.02±0.01 | 0.739 |
| urine PH | 5.5±0.8 | 5.4±0.7 | 5.5±0.8 | 0.071 |
| **Medications and device therapies** |  |  |  |  |
| Ablation，n，% | 13(1.3) | 1(0.5) | 12(1.5) | 0.483 |
| CRT or ICD，n，% | 52(5.4) | 5(2.7) | 47(6) | 0.073 |
| PCI or CABG，n，% | 174(18) | 24(13) | 150(19.2) | 0.048 |
| Loop diuretic，n，% | 930(96.2) | 185(100) | 745(95) | ＜0.001 |
| ACEI/ARB，n，% | 692(71.6) | 126(68.1) | 566(72.4) | 0.247 |
| Antisterone，n，% | 909(94) | 177(95.7) | 732(93.6) | 0.286 |
| Beta Blocker，n，% | 645(66.7) | 102(55) | 543(69) | ＜0.001 |
| CCB，n，% | 118(12) | 19(10.3) | 99(12.7) | 0.372 |
| Antiplatelet，n，% | 704(72.8) | 138(74.6) | 566(72.4) | 0.542 |
| Anticoagulant，n，% | 265(27.4) | 44(23.8) | 221(28.3) | 0.220 |
| Statin，n，% | 625(64.6) | 114(61.6) | 511(65.3) | 0.341 |
| Positive inotropic drugs，n，% | 656(67.8) | 140(75.7) | 516(66) | 0.011 |
| Venous dilatation，n，% | 586(60.6) | 121(65.4) | 456(58.3) | 0.137 |
| Oral anti-arrhythmic，n，% | 223(23.1) | 51(27.6) | 172(22) | 0.106 |
| Antibiotic，n，% | 573(59.3) | 136(73.5) | 437(55.9) | ＜0.001 |
| CN medicine，n，% | 72(7.4) | 17(9.2) | 55(7) | 0.315 |
| Nutritional myocardium drugs，n，% | 156(16.1) | 26(14.1) | 130(16.6) | 0.393 |
| **Echocardiographic** |  |  |  |  |
| LA(mm) | 48.8±8.8 | 49.6±9.1 | 48.7±8.7 | 0.212 |
| LVEF(%) | 45(33,60) | 43(32,59) | 45(34,61) | 0.127 |
| FS(%) | 24.6±9.9 | 23.5±9.4 | 24.9±10.0 | 0.085 |
| LVEDD(mm) | 62.5±11.4 | 62.9±11.0 | 62.4±11.5 | 0.626 |
| LVESD(mm) | 48.0±13.5 | 48.8±13.0 | 47.8±13.7 | 0.386 |
| SPAP(mmHg) | 42(32,54) | 42(33,54) | 42(32,54) | 0.248 |
| TRPG(mmHg) | 34(24,45) | 34(25,45) | 33(24,45) | 0.173 |
|  |  |  |  |  |
|  |  |  |  |  |

Abbreviations as in Table S1
